# Rebalancing Redox to Improve Biobutanol Production by *Clostridium tyrobutyricum*

**DOI:** 10.3390/bioengineering3010002

**Published:** 2015-12-24

**Authors:** Chao Ma, Jianfa Ou, Ningning Xu, Janna L. Fierst, Shang-Tian Yang, Xiaoguang (Margaret) Liu

**Affiliations:** 1 Department of Chemical and Biological Engineering, The University of Alabama, 245 7th Avenue, Tuscaloosa, AL 35401, USA; cma3@crimson.ua.edu (C.M.); jou4@crimson.ua.edu (J.O.); nxu2@crimson.ua.edu (N.X.); 2 Department of Biological Science, The University of Alabama, 300 Hackberry Lane, Tuscaloosa, AL 35487, USA; janna.l.fierst@ua.edu; 3 Department of Chemical and Biomolecular Engineering, The Ohio State University, 151 West Woodruff Avenue, Columbus, OH 43210, USA; yangst.osu.2013@gmail.com

**Keywords:** *Clostridium tyrobutyricum*, butanol production, redox engineering, metabolic cell engineering, metabolic shift

## Abstract

Biobutanol is a sustainable green biofuel that can substitute for gasoline. Carbon flux has been redistributed in *Clostridium tyrobutyricum* via metabolic cell engineering to produce biobutanol. However, the lack of reducing power hampered the further improvement of butanol production. The objective of this study was to improve butanol production by rebalancing redox. Firstly, a metabolically-engineered mutant CTC-*fdh*-*adhE2* was constructed by introducing heterologous formate dehydrogenase (*fdh*) and bifunctional aldehyde/alcohol dehydrogenase (*adhE2*) simultaneously into wild-type *C. tyrobutyricum*. The mutant evaluation indicated that the *fdh*-catalyzed NADH-producing pathway improved butanol titer by 2.15-fold in the serum bottle and 2.72-fold in the bioreactor. Secondly, the medium supplements that could shift metabolic flux to improve the production of butyrate or butanol were identified, including vanadate, acetamide, sodium formate, vitamin B12 and methyl viologen hydrate. Finally, the free-cell fermentation produced 12.34 g/L of butanol from glucose using the mutant CTC-*fdh*-*adhE2*, which was 3.88-fold higher than that produced by the control mutant CTC-*adhE2*. This study demonstrated that the redox engineering in *C. tyrobutyricum* could greatly increase butanol production.

## 1. Introduction

n-Butanol is a potential substitute for gasoline, a raw material to generate bio-jet fuel and an important industrial chemical. Biobutanol has been produced by solventogenic Clostridia, such as *C. acetobutylicum* and *C. beijerinckii*, in acetone-butanol-ethanol (ABE) fermentation [1,2]. Extensive metabolic engineering and fermentation development have improved butanol production [3,4,5,6]. However, ABE fermentation still suffers from the relatively low butanol yield, titer and productivity due to the butanol inhibition and the complicated metabolic pathway involved in acidogenesis, solventogenesis and sporulation. 

One alternative strategy for biobutanol production is to synthesize a heterologous butanol biosynthesis pathway from solventogenic Clostridia in other microbes, including *Escherichia coli*, *Saccharomyces cerevisiae*, *Pseudomonas putida*, *Bacillus subtilis* and *Lactobacillus brevis* [7,8,9,10,11,12]. For instance, integrated synthetic biology, carbon engineering and redox engineering have been performed in *E. coli,* which produced 14 g/L of n-butanol [13]. Despite these achievements, the inability to ferment low-cost feedstock, the low efficiency to catabolize the hydrolysate of biomass, the low butanol tolerance or the low butanol productivity by these mutants has limited their industrial applications in biobutanol production. 

*C. tyrobutyricum*, an anaerobic acidogenic strain naturally producing butyrate, acetate, CO_2_ and H_2_ [14,15], is a promising bacterium for biobutanol production due to its advantages of high butanol tolerance, relatively simple metabolic pathway and high conversion rate of butyryl-CoA from sugars [16,17,18]. Recently, the butanol-producing mutants CTC-*adhE2* and ACKKO*-adhE2* were developed by expressing a bifunctional aldehyde/alcohol dehydrogenase (*adhE2*) from *C. acetobutylicum* ATCC 824 in wild-type and high butyrate-producing mutants of *C. tyrobutyricum*, respectively. The free-cell fermentation produced 2.5–6.5 g/L of butanol by CTC-*adhE2* and 14.5–16 g/L of butanol by ACKKO*-adhE2* from glucose [16,17]. Advanced fermentation processes, such as using mannitol as a substrate or immobilizing mutant cells in a fibrous bed bioreactor supplemented with methyl viologen hydrate, were also developed to further improve biobutanol titer to 20 g/L [16,18]. 

Although rebalancing carbon resulted in high butanol production by *C. tyrobutyricum*, the lack of reducing power is still a challenge to further improve biobutanol production [17,19]. Redox engineering has been applied to improve the accumulation of NADH in *E. coli*, *S. cerevisiae* and *Klebsiella pneumonia* [10,13,20,21,22]. For example, the overexpression of formate dehydrogenase (*fdh*) improved biobutanol titer from 0.2 g/L–0.52 g/L by *E. coli* and from ~0.6 g/L–0.9 g/L by *K. pneumonia* [10,22]. The redox engineering via introducing Fdh into *C. tyrobutyricum* has not been evaluated so far [19]. 

**Figure 1:**
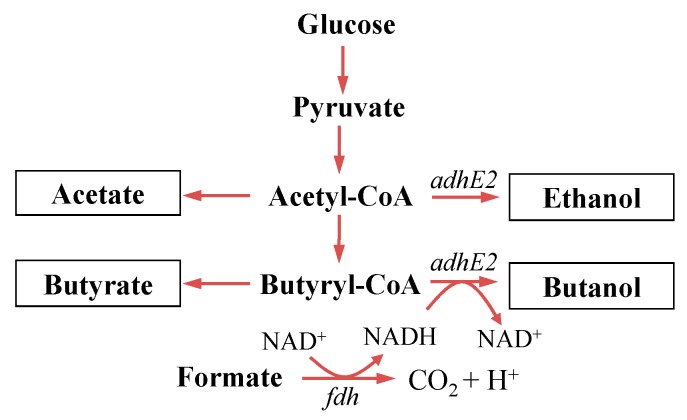
Butanol producing metabolic pathway in *C. tyrobutyricum*. The wild-type CTC produces butyrate and acetate; mutant CTC-*adhE2* produces butanol and ethanol via carbon rebalance by overexpressing the *adhE2* gene [16]; and mutant CTC-*fdh*-*adhE2* constructed in this study shows improved butanol production via redox rebalance by overexpressing the *fdh* gene.

The objective of this study was to improve biobutanol production via rebalancing redox in *C. tyrobutyricum* (Figure 1). Redox engineering was performed to construct a new mutant CTC-*fdh-adhE2*, and the components that boosted its butanol production were also identified. The free-cell butanol fermentation indicated that redox rebalance, in addition to carbon rebalance, is an efficient approach to significantly improve butanol production. 

## 2. Materials and Methods

### 2.1. Strains and Media

As listed in Table 1, wild-type, mutant CTC-*adhE2* and mutant CTC-*fdh-adhE2* of *C. tyrobutyricum* ATCC 25755 were used. The wild-type strain was purchased from ATCC (ATCC, Manassas, VA, USA). The control mutant (CTC-*adhE2*) was obtained from Yang’s lab [23]. In this study, the mutant CTC-*fdh-adhE2* was constructed by simultaneously expressing the heterologous formate dehydrogenase gene (*fdh*) and bifunctional aldehyde/alcohol dehydrogenase (*adhE2*) in wild-type *C. tyrobutyricum*. 

**Table 1:** Strains and plasmids used in this study.

Plasmid/Strain	Relevant Characteristics	Reference/Source
**Plasmids**		
*p*MTL-adhE2	*adhE2* overexpression with *thl* promoter	This study
*p*MTL-fdh-adhE2	*fdh* and *adhE2* overexpression with *thl* promoter	This study
**Strains**		
*C. tyrobutyricum*	Clostridium, ATCC 25755, wild-type	ATCC
CTC-*adhE2*	Clostridium with *adhE2* overexpression, thiamphenicol resistant	[23]
CTC-*fdh-adhE2*	Clostridium with *fdh* and *adhE2* overexpression, thiamphenicol resistant	This study
*E. coli* CA434	*E. coli* HB101 with plasmid R702, kanamycin resistant	[24]

The seed culture of *C. tyrobutyricum* was maintained anaerobically in reinforced Clostridial medium (RCM; Difco, Kansa City, MO, USA). The 30 μg/mL of thiamphenicol (Tm, Alfa Aesar, Ward Hill, MA, USA) was used to select and maintain mutants. In serum bottle and bioreactor fermentations, the modified Clostridial growth medium (CGM) containing 40 g/L of glucose was used [25]. Antibiotics were added to the baseline study in the serum bottle, but not added to media study and bioreactor fermentation. *E. coli* was grown aerobically in Luria–Bertani (LB) media supplemented with 30 μg/mL of chloramphenicol (Cm, Alfa Aesar) or 50 μg/mL of kanamycin (Kan, Alfa Aesar). The chemical reagents were purchased from Sigma-Aldrich (St. Louis, MO, USA), unless otherwise specified. 

### 2.2. Plasmid Construction

**Figure 2:**
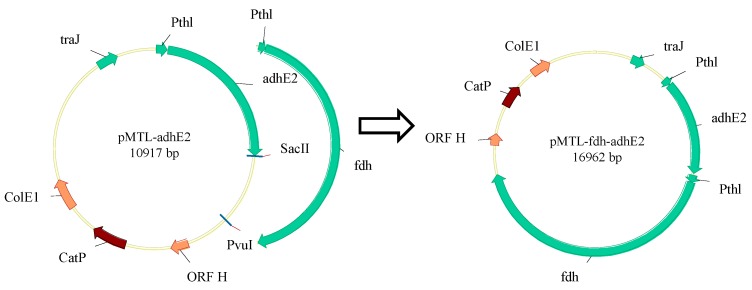
Plasmid construction. *ORF H*: *pCB102* replicon from *Clostridium butyricum*; *CatP*: chloramphenicol- and thiamphenicol-resistant gene; *ColE1*: *E. coli* replicon; *fdh*: formate dehydrogenase from *Moorella thermoacetica*; *Pthl*: promoter of thiolase from *Clostridium tyrobutyricum*; *traJ*: regulator of the F plasmid transfer operon; *adhE2*: bifunctional aldehyde/alcohol dehydrogenase (*adhE2*) from *Clostridium acetobutylicum*.

The flowchart of plasmid construction is described in Figure 2. Specifically, the pMTL007 plasmid obtained from Minton’s lab [26] was used as an expression vector. The *fdh* gene was amplified from *Moorella thermoacetica* ATCC 39073 (ATCC, Manassas, VA, USA) using Q5 High-Fidelity DNA Polymerase (NEB, Ipswich, MA, USA). The plasmid pMTL-*adhE2* was constructed following a previous publication [23]. The promoter of the homologous *thl* gene (Pthl) cloned from *C. tyrobutyricum*, the *fdh* gene and pMTL-*adhE2* backbone were assembled to generate the plasmid pMTL-*fdh-adhE2* using the Gibson Assembly kit (NEB) following the manufacture’s instruction. 

### 2.3. Mutant Construction

The transformation of plasmid pMTL-*fdh-adhE2* into the wild-type *C. tyrobutyricum* was performed via conjugation in an anaerobic chamber according to a previous publication [23,24] with the following modifications. The *E. coli CA434* [24] was transformed with pMTL-*fdh-adhE2* and cultivated in LB medium containing 30 μg/mL of Cm and 50 μg/mL of Kan. The *E. coli CA434*/pMTL-*fdh-adhE2* was harvested as donor cells when the optical density at 600 nm (OD_600_) reached ~1.5. The wild-type cells were grown in RCM medium and collected as recipient cells when OD_600_ reached 1.5–3.0. The transformed clostridial cells were spread on RCM selection plates that contained 30 μg/mL of Tm and 250 μg/mL of D-cycloserine (Cy, Alfa Aesar). The selection plates were incubated at 37 °C for 72–96 h or until colonies appeared. Twenty colonies were picked and evaluated in a 50-mL serum bottle culture to screen the clone with the highest butanol production, which was named CTC-*fdh-adhE2*. 

### 2.4. Medium Supplements Screening

To engineer the butanol fermentation process, medium optimization was performed in the small-scale fermentations of CTC-*fdh-adhE2* in serum bottles. In this study, ten components at two levels (*i.e.*, concentrations) were analyzed using the new mutant CTC-*fdh-adhE2*. Only the representative conditions that enhanced the production of butyrate or butanol are summarized in Table 2. Fresh seed culture with an OD_600_ of ~1.5 was used to inoculate the 50 mL CGM medium containing 40 g/L of glucose and different medium supplements. The cultures with inoculation OD_600_ of ~0.04 were anaerobically incubated at 37 °C without pH adjustment. The samples were taken daily to monitor cell growth, glucose consumption and product formation. All conditions were carried out in duplicate, and data are reported as means with standard deviations.

**Table 2:** Medium component screening to rebalance redox and carbon.

No.	Sodium Formate (1 g/L)	Vitamin B12 (0.001 g/L)	Methyl Viologen Hydrate (0.1 g/L)	Vanadate (1 g/L)	Acetamide (1 g/L)
C	−	−	−	−	−
1	+	−	−	−	−
2	−	+	−	−	−
3	−	−	+	−	−
4	+	+	−	−	−
5	+	−	+	−	−
6	+	+	+	−	−
7	+	−	−	+	−
8	+	−	−	−	+
9	+	+	−	+	−
10	+	+	−	−	+

### 2.5. Butanol Fermentation

The kinetics studies of the fed-batch fermentation by CTC-*adhE2* (control) and CTC-*fdh-adhE2* mutants were performed in a stirred-tank bioreactor (FS-01-A; Major science, Saratoga, CA, USA). Fermentation setup, operation and sampling were performed as reported previously [17]. Fermentations were run in duplicate, and the means and standard deviations were calculated. 

### 2.6. Activity Assay of Formate Dehydrogenase 

The preparation of cell extract and the measurement of formate dehydrogenase activity were performed as reported with modification [27,28]. The cells of CTC-*adhE2* and CTC-*fdh-adhE2* were collected when the OD_600_ reached ~2.0, centrifuged, washed and cooled in an anaerobic chamber. The suspended cells on ice were sonicated for 15 min using a sonifier (Models 250; Branson Ultrasonics, Danbury, CT, USA) with 40% power and a 30 s interval for cooling each minute. The concentration of total protein in cell extract was titrated using Bradford’s kit (Bio-Rad, Hercules, CA, USA) with bovine serum albumin as the standard. 

The activity assay of formate dehydrogenase was performed at 37 °C in an anaerobic chamber. Specifically, 0.1 mL of cell extract was added to 1 mL of pre-mixed potassium phosphate buffer containing 1.67 mM of NAD^+^ and 167 mM of sodium formate. The kinetics of NADH generation was monitored by measuring the absorbance at 340 nm at the reaction times of 10, 20, 30, 60, 90 and 120 s using a spectrophotometer (Biomate3; Thermo Fisher Scientific, Hudson, NH, USA). The concentration of NADH was calculated using a specific absorbance coefficient of 6220 M^−1^·cm^−1^. One unit of the activity of formate dehydrogenase was defined as 1 μmol of NADH produced per minute at pH 7.5 and 37 °C. The specific activity of Fdh was calculated from the measured activity divided by total protein in the cell extract. 

### 2.7. Butanol Tolerance

To evaluate the effect of redox engineering on butanol tolerance, the wild-type, CTC-*adhE2* and CTC-*fdh-adhE2* were cultivated in serum bottles with a seeding OD_600_ of 0.3. Butanol at final concentrations of 0, 5, 10, 15 and 20 g/L was added to the basal CGM medium. The cell growth was analyzed by sampling serum bottles every 3 h. The butanol inhibition was modeled by the equation of μ = μ_max_Kp/(Kp + P), where μ is the specific growth rate (h^−1^), Kp is the inhibition rate constant (g/L) and P is the butanol concentration (g/L). 

### 2.8. NADH Assay

An NAD/NADH quantitation colorimetric kit (BioVision, San Francisco, CA, USA) was used to measure the intracellular concentration of NADH and NAD^+^ in the CTC, CTC-*adhE2* and CTC-*fdh-adhE2*. The cells were cultivated in 50 mL CGM medium containing 1 g/L of sodium formate in serum bottles with duplication. The cultures were sampled in mid-log phase, and 1-mL samples were centrifuged at 10,000 rpm for 10 min. The cell pellets were re-suspended in 400 μL of NADH/NAD extraction buffer provided in the kit. The NADH and NAD^+^ were extracted by freeze/thaw for two cycles (20 min on dry-ice followed by 10 min at room temperature in each cycle). To titrate NADH, NAD^+^ was decomposed by heating the cell lysate at 60 °C by 30 min. After cooling on ice for 10 min, the NADH samples were centrifuged at 4000 rpm for 2 min. The 50 μL of supernatant were mixed with 100 µL of NAD^+^ cycling enzyme provided in the kit. The reaction mixture was incubated at room temperature for 4 h before the value of OD_450_ was read. To measure the concentration of NAD^+^ and NADH, 4 μL of cell lysate was mixed with 46 µL of NADH/NAD extraction buffer and 100 µL of NAD cycling enzyme. The NADH concentration was calculated using the equation “Concentration_NADH_ (mM) = Mole_NADH_ /Volume_wet cell_ = Mole_NADH_ /(OD_600_/mL × 0.38 g-dry cell/L × 10^−3^ L/mL × 4.27 mL/g-wet cell × 10^−3^ g/mg)”.

## 3. Analytical Methods

The cell density was analyzed by measuring the OD_600_ of the cell suspension. The concentrations of fermentation substrate and products, including glucose, butanol, butyrate, acetate and ethanol, were analyzed using high performance liquid chromatography (HPLC, Shimadzu, Columbia, MD, USA) following previously-published methods [17]. The glucose concentration was also analyzed daily using a YSI 2700 Select Biochemistry Analyzer (YSI Life Sciences, Yellow Springs, OH, USA) to determine the feeding strategy.

## 4. Results 

### 4.1. Construction and Evaluation of Redox-Engineered Mutant

In this study, the heterologous formate dehydrogenase gene (*fdh*) was overexpressed together with *adhE2* in wild-type *C. tyrobutyricum* to generate the new mutant CTC-*fdh-adhE2*. The enzyme AdhE2 catalyzed the formation of butanol from butyryl-CoA, which had been demonstrated in the previous study [23]. In this study, the enzyme Fdh was expressed to rebalance redox by generating more reducing power, *i.e.*, NADH. 

**Figure 3:**
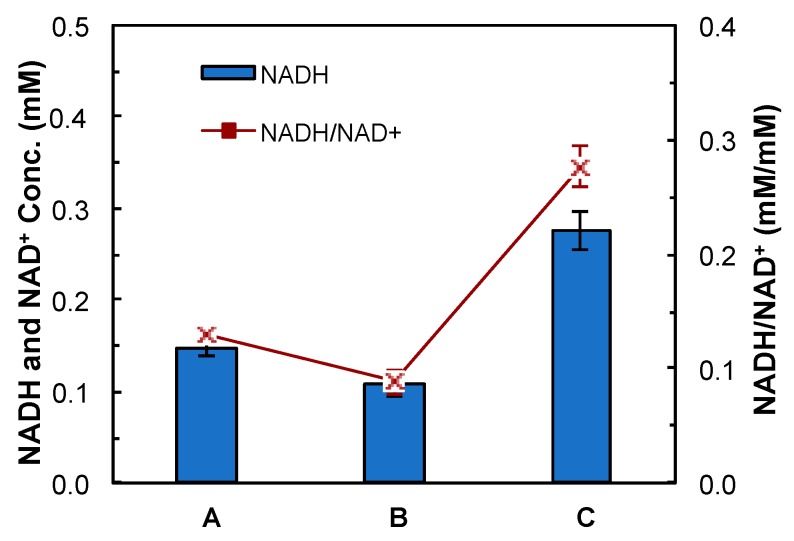
NADH assay. A: CTC wild-type; B: CTC-*adhE2*; and C: CTC-*fdh-adhE2*. ■: mole ratio between NADH and NAD^+^. Conc.: concentration.

The mutant CTC-*fdh-adhE2* was characterized using PCR, an enzyme activity assay and a NADH assay. The heterologous *fdh* gene was successfully amplified from CTC-*fdh-adhE2* in the PCR reaction, indicating that the pMTL-*fdh-adhE2* plasmid was transformed into *C. tyrobutyricum*. The enzyme assay showed that the specific enzymatic activity of the NAD^+^-dependent Fdh was 990 U/g in CTC-*fdh-adhE2* and zero in CTC-*adhE2*. These results confirmed that the heterologous fdh was expressed properly in the CTC-*fdh-adhE2*. In addition, the NADH assay demonstrated that the intracellular NADH concentration was increased from 0.11 mM in CTC-*adhE2* to 0.28 mM in CTC-*fdh-adhE2*, as shown in Figure 3. The mole ratio between NADH and NAD^+^ was also enhanced in CTC-*fdh-adhE2*. These results confirmed that the synthesized Fdh enzyme boosted the intracellular accumulation of NADH. 

**Figure 4:**
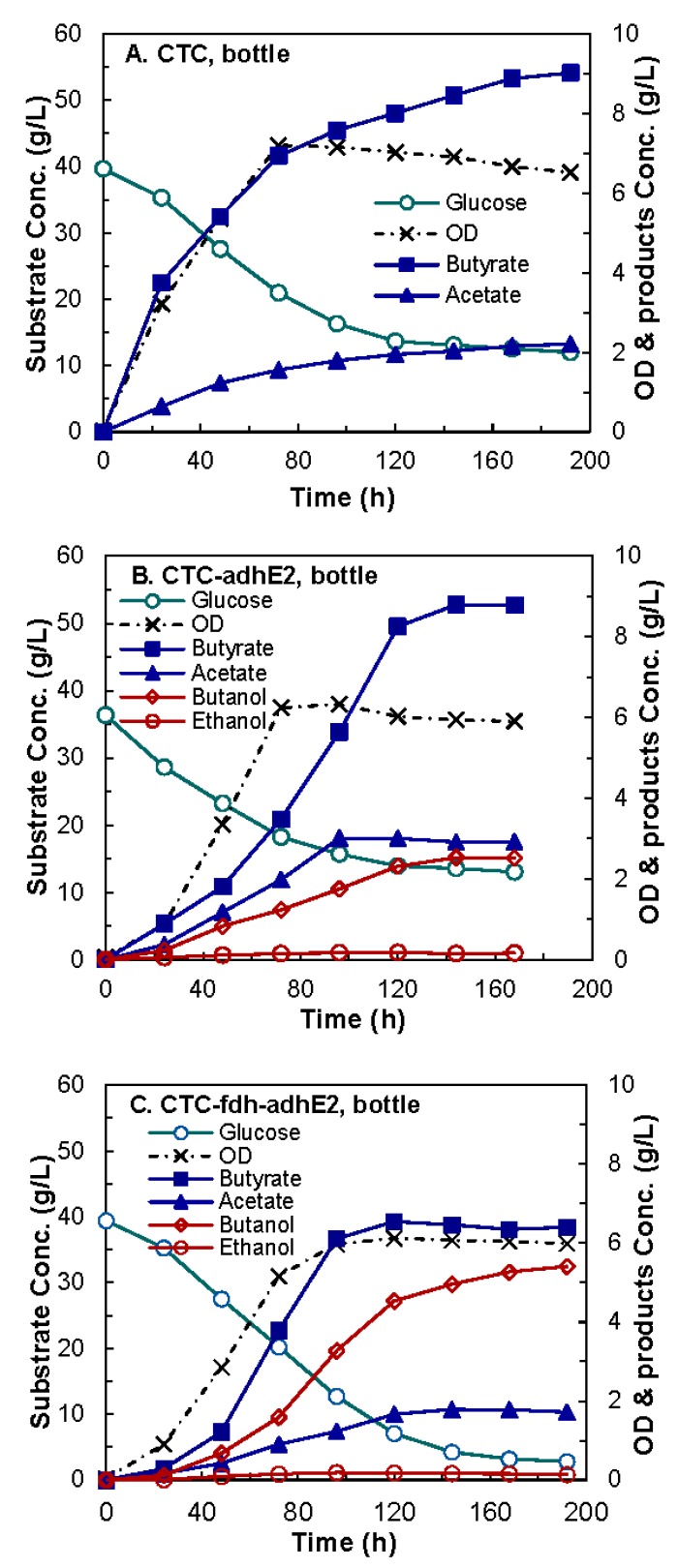
Kinetics butanol fermentation by *C. tyrobutyricum* in serum bottles. (**A**) CTC wild-type; (**B**) CTC-*adhE2*; and (**C**) CTC-*fdh-adhE2*. ○: glucose; ×: OD; ■: butyrate; ▲: acetate; ◊: butanol; ○: ethanol.

The baselines of butanol production of wild-type (Control 1), CTC-*adhE2* (Control 2), and CTC-*fdh-adhE2* were created using a 100-mL culture in serum bottles. The modified CGM medium was used without the addition of formate. As presented in the kinetics profiles in Figure 4, all three cells grew immediately after inoculation and reached a similar maximum OD_600_. The fermentation data showed that the wild-type produced 9.02 g/L of butyrate and no butanol, as expected. The CTC-*adhE2* produced 8.78 g/L of butyrate and 2.51 g/L of butanol, and the CTC-*fdh*-*adhE2* produced 6.40 g/L of butyrate and 5.41 g/L of butanol. The CTC-*fdh-adhE2* also produced 2.15-fold higher butanol than CTC-*adhE2*. These results indicated that the overexpression of the heterologous gene *fdh* in CTC-*fdh-adhE2* shifted carbon flux from butyrate to butanol. 

The butanol production consumes reducing power NAD(P)H in *C. tyrobutyricum*, and it is important to evaluate the feasibility of improving butanol production via rebalancing redox. In this study, we introduced an NADH-producing pathway by overexpressing an NAD^+^-dependent enzyme Fdh in CTC-*adhE2*. NADH can be produced from formate through the synthesized reaction “Formate + NAD^+^ ⇋ NADH + CO_2_ + H^+^”. The increase of butanol production by CTC-*fdh-adhE2* indicated that the expression of Fdh generated more NADH and, thus, benefited butanol production.

In addition, it was noted that the butanol production was improved by CTC-*fdh-adhE2* even though no additional formate was added to the medium. Our proteomics study showed that pyruvate-formate lyase (*pfl*) was expressed in *C. tyrobutyricum* (data not shown). The enzyme *pfl* catalyzes the reversible reaction “pyruvate + CoA ⇋ formate + acetyl-CoA”. Because pyruvate was produced from glucose through the glycolysis pathway, formate could be intracellularly produced from pyruvate. The generated formate could be consumed by Fdh to boost the NADH and improve butanol production. 

### 4.2. Evaluation of Medium Supplements

**Figure 5:**
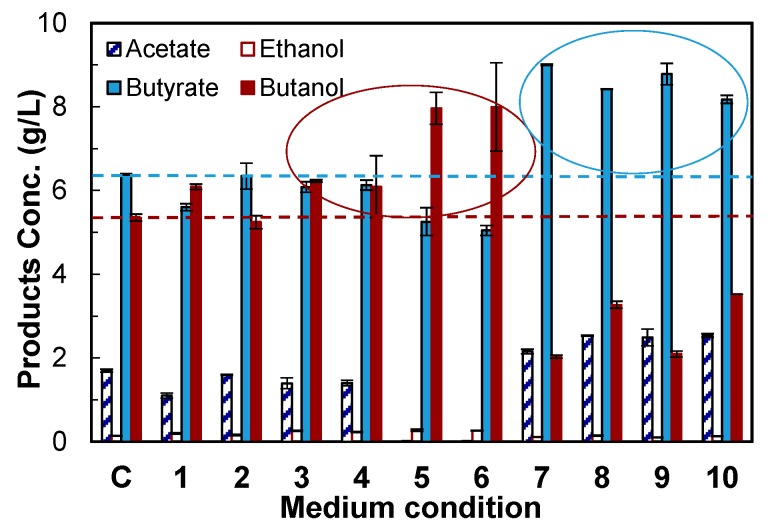
Screening of medium components to rebalance carbon and redox flux in CTC-*fdh-adhE2*. C: control without medium supplement; 1: + sodium formate (formate); 2: + vitamin B12 (B12); 3: + methyl viologen hydrate (MVH); 4: + formate + B12; 5: + formate + MVH; 6: + formate + B12 + MVH; 7: + formate + vanadate; 8: + formate + acetamide; 9: + formate + B12 + vanadate; and 10: + formate + B12 + acetamide. □: acetate; □: ethanol; ■: butyrate; ■: butanol. The experimental design is given in Table 2. The data are the means of the replicated fermentations in serum bottles.

Medium optimization, an important process engineering strategy, was carried out to screen the components that could increase the formation of butanol. The average data of the final titers of acetate, butyrate, ethanol and butanol by CTC-*fdh-adhE2* are described in Figure 5. The control culture without any medium supplement produced 5.35 g/L of butanol. Some components increased butanol up to 8.00 g/L, but some components increased butyrate up to 9.00 g/L. The ten experimental conditions were classified into two groups (Table 2 and Figure 5), including one that improved butanol production and a second that enhanced butyrate production. 

In the first group, either sodium formate (SF, 1 g/L, Condition 1) or methyl viologen hydrate (MVH, 0.1 g/L, Condition 3) improved the butanol production. As discussed above, formate increased butanol production because the Fdh enzyme synthesized more NADH. MVH is an artificial electron carrier that can boost electron supply from ferredoxin, so it increased butanol production by providing more reducing power. MVH was also used to improve butanol production by immobilized *C. tyrobutyricum* in a recent study [18]. A previous study reported that B12 improved the cell growth of *C. acetobutylicum* [29], but did not investigate the effect of B12 on butanol production. This study showed that vitamin B12 (B12, 0.001 g/L) did not change butanol production (Condition 2), although it improved the maximum OD by 36% compared to the control. 

The combination of formate, B12 and MVH (Condition 6) improved the butanol titer to 8.00 g/L and reduced the butyrate titer to 5.05 g/L compared to the control condition. The selectivity of butanol was also improved from 0.39 g/g (control) to 0.60 g/g. Interestingly, the supplement of these three components significantly reduced the acetate production to 0.01 g/L and improved C4 (butyrate and butanol) selectivity to 0.97 g/g, while the control culture produced acetate with a titer of 1.70 g/L and C4 (butyrate and butanol) with a selectivity of 0.87 g/g. Taken together, the addition of formate, B12 and MVH to CGM medium not only shifted more carbon flux from butyrate to butanol, but also redirected carbon from C2 to C4. We also observed that the addition of any of these three components (Conditions 1–3) did not reduce the acetate formation, but the combination of formate and MVH (Conditions 5 and 6) blocked the production of acetate. We hypothesized that a synergistic effect between formate and MVH shifted carbon flux from C2 to C4. 

In the second group, the addition of vanadate and acetamide increased the production of butyrate and acetate. Acetamide could provide an extra nitrogen source, and vanadate could affect the metabolism of sugar phosphate. Both components shifted carbon from butanol to butyrate. Other key findings that are not described in Table 2 or Figure 5 include: (1) sodium pyruvate (1 g/L) did not increase the production of solvents and acids; and( 2) manganese sulfate (0.1 g/L) increased acetate production (2.55 *vs*. 1.70 g/L by the control) because the high activity of acetate kinase needs manganese [30]. 

Very interestingly, both butanol and ethanol formation pathways consumed NADH, but neither the expression of Fdh nor the medium supplements improved the production of ethanol, *i.e.*, 0.28 g/L in Condition 6 supplemented with formate, B12 and MVH *vs.* 0.14 g/L in the control. Our previous proteomics study of *C. tyrobutyricum* showed that the expression of alcohol dehydrogenase (*adh*) was very low, while the expression of butanol dehydrogenase (*bdh*) was pretty high [17]. Therefore, the redox engineering increased the production of butanol, but not ethanol due to the low expression of *adh* and the low metabolic flux distributed to ethanol. 

### 4.3. Butanol Fermentation 

**Figure 6:**
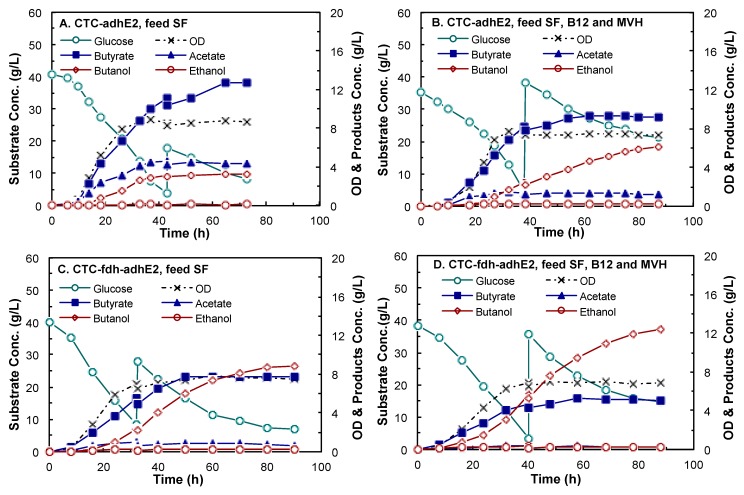
Kinetics of butanol fermentation by *C. tyrobutyricum* in 2-L bioreactors at pH 6.0, 37 °C and 100 rpm. (**A**) CTC-*adhE2* with sodium formate (SF); (**B**) CTC-*adhE2* with sodium formate, vitamin B12 (B12) and methyl viologen hydrate (MVH); (**C**) CTC-*fdh-adhE2* with SF; and (**D**) CTC-*fdh-adhE2* with SF, B12 and MVH. ○: glucose; ×: OD; ■: butyric acid; ▲: acetic acid; ◊: butanol; ○: ethanol.

Both CTC-*adhE2* and CTC-*fdh-adhE2* were evaluated in 2-L fed-batch fermentations in a stirred-tank bioreactor at 37 °C and pH 6.0 using CGM medium supplemented with the identified components. As shown in Figure 6, four conditions were investigated, including CTC-*adhE2* supplemented with SF (Figure 6A), CTC-*adhE2* with sodium formate (SF), vitamin B12 (B12) and methyl viologen hydrate (MVH) (Figure 6B), CTC-*fdh-adhE2* with SF (Figure 6C) and CTC-*fdh-adhE2* with SF, B12 and MVH (Figure 6D). The kinetics profiles in Figure 5 showed that there was no obvious lag phase in all fermentations. As presented in Table 3, all four conditions had similar specific growth rates of 0.15–0.18 h^−1^ and a maximum OD_600_ of 7.5–9.0. 

**Table 3:** Butanol fermentations using metabolically-engineered *C. tyrobutyricum* by rebalancing redox.

Products		CTC-*adhE2* (Control)		CTC-*fdh-adhE2*	
		SF	SF, B12 and MVH	SF	SF, B12 and MVH	
Cell growth (h^−1^)		0.18 ± 0.01	0.16 ± 0.0004	0.15 ± 0.001	0.15 ± 0.001	
Biomass yield (g/g)		0.10 ± 0.01	0.09 ± 0.003	0.09 ± 0.001	0.08 ± 0.002	
Concentration (g/L)	Butanol	3.18 ± 0.09	6.14 ± 0.05	8.83 ± 0.02	12.34 ± 0.02	
	Butyrate	13.22 ± 0.73	9.32 ± 0.03	7.72 ± 0.05	5.05 ± 0.04	
	Acetate	4.15 ± 0.25	1.42 ± 0.07	0.69 ± 0.01	0.26 ± 0.03	
	Ethanol	0.11 ± 0.04	0.25 ± 0.02	0.24 ± 0.01	0.28 ± 0.07	
Yield (g/g-glucose)	Butanol	0.05 ± 0.01	0.11 ± 0.003	0.16 ± 0.002	0.23 ± 0.002	
	Butyrate	0.27 ± 0.02	0.20 ± 0.004	0.19 ± 0.01	0.10 ± 0.001	
	Acetate	0.06 ± 0.01	0.02 ± 0.002	0.02 ± 0.001	0.003 ± 0.0001	
	Ethanol	0.001 ± 0.0005	0.003 ± 0.0004	0.004 ± 0.0002	0.004 ± 0.0003	
Productivity (g/L/h)	Butanol	0.12 ± 0.01	0.14 ± 0.01	0.17 ± 0.002	0.26 ± 0.01	
	Butyrate	0.33 ± 0.01	0.26 ± 0.003	0.22 ± 0.004	0.15 ± 0.004	
	Acetate	0.14 ± 0.01	0.07 ± 0.004	0.05 ± 0.01	0.01 ± 0.002	
	Ethanol	0.006 ± 0.001	0.008 ± 0.0001	0.010 ± 0.0003	0.011 ± 0.0004	

Notes: The means in this table were calculated from the duplicated fermentations. Yield = g-product/g-glucose consumed; and selectivity = g-product/g-total products. SF: sodium formate; B12: vitamin B12; MVH: methyl viologen hydrate.

As summarized in Table 3, the butanol concentrations were 3.18 g/L, 6.14 g/L, 8.83 g/L and 12.34 g/L by CTC-*adhE2* with SF, CTC-*adhE2* with SF, B12 and MVH, CTC-*fdh-adhE2* with SF and CTC-*fdh-adhE2* with SF, B12 and MVH, respectively. It is obvious that the integration of metabolic cell engineering (Fdh synthesis) and process engineering (medium optimization) significantly increased the butanol production from 3.18 g/L–12.34 g/L, with 3.88-fold improvement. The yield of butanol through integrated cell-process engineering (0.23 g/g) was also much higher than other conditions (0.05–0.16 g/g). The butanol production by redox engineering was slightly higher than that by medium supplement addition, *i.e.*, titer of 8.83 g/L and yield of 0.16 g/g (Figure 5C) *vs.* titer of 6.14 g/L and yield of 0.11 g/g (Figure 5B). However, butanol productivity was 0.26 g/L/h by CTC-*fdh*-*adhE2* with medium supplements, which was much higher than other conditions (0.12–0.17 g/L/h). These results confirmed that the rebalance of redox directed more carbon to the production of butanol that consumed reducing power. In addition, the ethanol concentrations in these four fermentations were fairly low (0.11 g/L–0.28 g/L) due to the low expression of alcohol dehydrogenase (*adh*).

**Figure 7:**
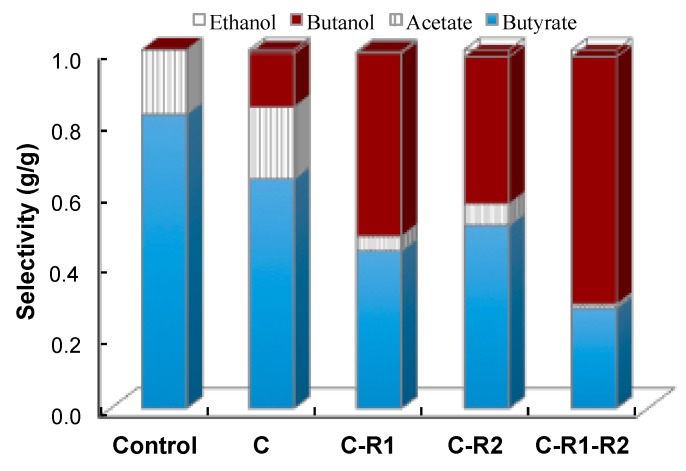
Improvement of n-butanol selectivity by rebalancing carbon and redox flux. Control: wild-type CTC without sodium formate; C: CTC-*adhE2* without sodium formate; C-R1: CTC-*adhE2* with sodium formate, vitamin B12 and methyl viologen hydrate; C-R2: CTC-*fdh-adhE2* with sodium formate; and C-R1-R2: CTC-*fdh-adhE2* with sodium formate, vitamin B12 and methyl viologen hydrate.

To better understand the effect of carbon and redox rebalance on butanol production, we compared the selectivity of fermentation products (Figure 7). The selectivity of butanol was 0 g/g, 0.15 g/g, 0.51 g/g, 0.41 g/g and 0.69 g/g at the conditions of control, CTC-*adhE2* (C), CTC-*fdh*-*adhE2* (C-R1), CTC-*adhE2* with medium supplement (C-R2) and CTC-*fdh*-*adhE2* with medium supplement (C-R1-R2), respectively. It is clear that the rebalances of carbon and redox via integrating metabolic cell and process engineering could effectively boost the selectivity of butanol and improve the production of butanol. In addition; the conditions of C, C-R1, C-R2 and C-R1-R2 produced butyrate with concentrations of 13.22 g/L, 7.72 g/L, 9.32 g/L and 5.05 g/L and the acetate with concentrations of 4.15 g/L, 0.69 g/L, 1.42 g/L and 0.26 g/L, respectively. These results revealed that rebalancing redox not only redistributed carbon between C4, but also redirected the carbon flux from C2 to C4. 

### 4.4. Butanol Tolerance

**Figure 8:**
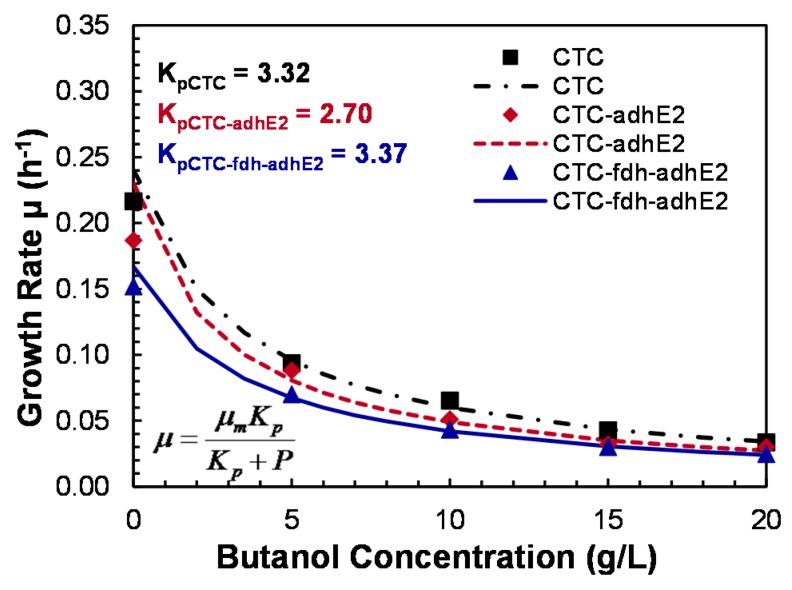
Butanol tolerance study of *C. tyrobutyricum* in serum bottles. ■: WT; -_∙_-_∙_-_∙_-: WT; ♦: CTC-*adhE2*; ------: CTC-adhE2; ▲: CTC-*fdh-adhE2*; **^_______^**: CTC-*fdh-adhE2*.

Butanol is a toxic solvent that could change the cell membrane fluidity and inhibit cell growth; butanol tolerance is therefore an important factor to evaluate in the new mutant. In this study, the effect of butanol on the cell growth of wild-type CTC, mutant CTC-*adhE2* and mutant CTC-*fdh*-*adhE2* was investigated (Figure 8). At 0 g/L of butanol, both mutants and the wild-type showed the highest specific growth rates, while higher butanol concentrations inhibited cell growth. At 20 g/L of butanol, less than 20% of the maximum growth rate was retained. The growth inhibition by butanol followed the noncompetitive inhibition kinetics with K_P_ (inhibition rate constant) of 3.32 g/L. 2.70 g/L and 3.37 g/L for CTC, CTC-*adhE2* and CTC-*fdh*-*adhE2*, respectively. Therefore, this tolerance study showed that metabolic engineering in the new mutant did not reduce butanol tolerance and cell growth. 

## 5. Discussion

### 5.1. Improvement of Butanol Production by Rebalancing Redox

In previous studies, it was found that the production of solvents involved a global remodeling of metabolism in *C. acetobutylicum* [31], and different NADH/NAD^+^ ratios could redistribute the metabolic flux in *E. coli* and *C. acetobutylicum* [20,32]. Our previous omics study also showed a strong correlation between the expression level of NAD(P)H-dependent enzymes and the production of butanol in *C. tyrobutyricum*, indicating that high butanol production required more reducing power [17]. Another study showed that the addition of methyl viologen hydrate increased the butanol production in fermentation [18]. Therefore, it was hypothesized that the integration of redox rebalance in the engineered mutant of *C. tyrobutyricum* with carbon rebalance could greatly improve the butanol production. 

To examine the above hypothesis, we performed redox engineering using both cell engineering and process engineering in this study. The control mutant is CTC-*adhE2*, *i.e.*, the wild-type *C. tyrobutyricum* containing synthesized *adhE2* [23]. Compared to CTC-*adhE2*, the redox engineering by cell engineering or process engineering (*i.e.*, medium optimization in this study) doubled the butanol production, while the integration of cell-process engineering quadrupled the butanol production (12.34 g/L *vs.* 3.18 g/L). Our results suggest that the integration of these two engineering methods effectively improved reducing power supply and rebalanced redox. Our results also demonstrated that redox engineering was essential to achieve high butanol production in addition to carbon engineering in *C. tyrobutyricum*. 

Another interesting observation from this study is that redox rebalance significantly reduced the production of acids (butyrate and acetate) by *C. tyrobutyricum*. Because butanol formation consumed NAD(P)H, the synthesis of the NADH-producing pathway and the medium supplements resulted in carbon redistribution from acetate and butyrate to butanol. Therefore, the selectivity of butanol was significantly increased in the fermentation of CTC-*fdh*-*adhE2* with medium supplements. Moreover, ethanol production was still low even though the butanol production was increased by redox rebalance. The high selectivity of butanol and low production of byproducts could benefit the separation of biobutanol and reduce the production cost of biofuel from bacterial fermentation. 

### 5.2. Comparison with Previous Studies 

As a promising host cell for butanol production, *C. tyrobutyricum* has the desired features, such as unassociated sporulation and cell autolysis, high butanol tolerance and the ability to convert low-value lignocellulosic biomass feedstock. Recently, two studies have sought to improve biobutanol production by *C. tyrobutyricum* through metabolic engineering or process engineering. As summarized in Table 4, the mutant CTC-*adhE2* with overexpressed *adhE2* and the pCB12 replicon in wild-type *C. tyrobutyricum* produced butanol with a titer of 2.5 g/L from glucose in a serum bottle in a previous study [23], and a similar result was obtained in this study. The pBP1 replicon improved butanol titer to 6.5 g/L from glucose in the serum bottle and further improved butanol titer to 20.0 g/L from mannitol in the bioreactor [16]. These results elucidated that the replicon optimization and substrate were very important for biobutanol production. Replicon optimization could be performed in our future metabolic engineering study. However, mannitol is an expensive raw material for butanol production, with a price of ~2000 U.S. dollars per ton, which prevents it from becoming an economically-competitive biobutanol in the fuel market. In this study, the redox was rebalanced by synthesizing a NADH-producing pathway in addition to carbon redistribution, resulting in a doubled butanol titer. The reducing power supplement further improved butanol production by quadrupling the titer to reach 12.3 g/L. Therefore, we can conclude that redox rebalance is an effective approach to improve the biobutanol production by *C. tyrobutyricum*. 

**Table 4:** Recent progresses of butanol production with *C. tyrobutyricum.*

Strain	Characteristics	Mode	Carbon	Titer (g/L)	Yield (g/g)	Productivity (g/L/h)	Reference
CTC-*adhE2*	*+adhE2*, pCB102 replicon	Bottle	Glucose	2.5	0.15 *	0.04 *	[16]
		Bottle	Glucose	2.6	0.14	0.03	This study
CTpM2	+*adhE2*, pBP1 replicon	Bottle	Glucose	6.5	0.24	0.20 *	[16]
		Bioreactor	Mannitol ^#^	20.0 ^#^	0.33 ^#^	0.32 ^#^	[16]
CTC-*fdh*-*adhE2*	*+fdh+adhE2*, pCB102 replicon	Bottle	Glucose	6.9	0.21	0.20	This study
		Bioreactor	Glucose	12.3	0.23	0.26	This study

Notes: * These data were estimated from the fermentation kinetic profile. ^#^ Mannitol is an expensive substrate that significantly increases the production cost of biobutanol.

Our recent butyrate fermentation using *C. tyrobutyricum* showed that the fermentation pH significantly affected the production of butyrate and cell growth [33]. Specifically, the immobilized-cell fermentations at pH 6.0, 6.5 and 7.0 produced butyrate with concentrations of 50.11 g/L, 63.02 g/L and 61.01 g/L. The metabolic flux analysis demonstrated that carbon flux from acetyl-CoA to acetoacetyl-CoA and the carbon flux from butyryl-CoA to butyrate were increased at pHs of 6.5 and 7.0 as compared to pH 6.0. In addition, the biomass yield of *C. tyrobutyricum* was also decreased at a higher pH. The pH values of the butanol fermentations in bioreactors were maintained at 6.0 in this study. Because butanol is produced from butyryl-CoA directly, the improved carbon flux from C2 to C4 at higher pH could benefit the formation of butanol. In the future study, we will optimize butanol fermentation by the constructed mutant CTC-*fdh*-*adhE2* in order to further improve butanol production. 

Other metabolic engineering strategies were used to increase butanol production by redistributing the metabolic flux from C2 to C4. For example, the ACKKO*-adhE2* achieved the highest concentration of butanol (14.5 g/L) in free-cell fermentation, and process engineering in immobilized-cell fermentation generated >20 g/L of butanol [18]. To achieve a higher butanol production, redox engineering could be applied in a high butanol-producing mutant, e.g., ACKKO-*adhE2*, in the future. To effectively integrate cell engineering and process engineering, metabolomics should be developed and applied to *C. tyrobutyricum*. Metabolic flux analysis modeling is also an effective tool to guide the design of metabolic engineering [33,34]. In addition, the medium components can be rationally designed to assist in butanol production post cell engineering.

## 6. Conclusions

In this study, we demonstrated that redox engineering was essential to improve butanol production in *C. tyrobutyricum*. Both the titer and selectivity of butanol were significantly improved by rebalancing redox in addition to rebalancing carbon. We also found that the integration of metabolic cell and process engineering was an efficient strategy to improve biofuel production. 
